# Immune Response of Inactivated Rabies Vaccine Inoculated via Intraperitoneal, Intramuscular, Subcutaneous and Needle-Free Injection Technology-Based Intradermal Routes in Mice

**DOI:** 10.3390/ijms241713587

**Published:** 2023-09-02

**Authors:** Huiting Zhao, Peixuan Li, Lijun Bian, Wen Zhang, Chunlai Jiang, Yan Chen, Wei Kong, Yong Zhang

**Affiliations:** 1 National Engineering Laboratory for AIDS Vaccine, School of Life Sciences, Jilin University, Changchun 130012, China; 2 Key Laboratory for Molecular Enzymology and Engineering of Ministry of Education, School of Life Sciences, Jilin University, Changchun 130012, China; 3 NMPA Key Laboratory of Humanized Animal Models for Evaluation of Vaccines and Cell Therapy Products, Jilin University, Changchun 130012, China

**Keywords:** rabies vaccine, inoculation route, needle-free injection technology, intradermal injection, intramuscular injection, subcutaneous injection, intraperitoneal injection

## Abstract

Inoculation routes may significantly affect vaccine performance due to the local microenvironment, antigen localization and presentation, and, therefore, final immune responses. In this study, we conducted a head-to-head comparison of immune response and safety of inactivated rabies vaccine inoculated via intraperitoneal (IP), intramuscular (IM), subcutaneous (SC) and needle-free injection technology-based intradermal (ID) routes in ICR mice. Immune response was assessed in terms of antigen-specific antibodies, antibody subtypes and neutralizing antibodies for up to 28 weeks. A live rabies virus challenge was also carried out to evaluate vaccine potency. The dynamics of inflammatory cell infiltration at the skin and muscle levels were determined via histopathological examination. The kinetics and distribution of a model antigen were also determined by using in vivo fluorescence imaging. Evidence is presented that the vaccine inoculated via the ID route resulted in the highest antigen-specific antibody and neutralizing antibody titers among all administration routes, while IP and IM routes were comparable, followed by the SC route. Antibody subtype analysis shows that the IP route elicited a Th1-biased immune response, while SC and IM administration elicited a prominent Th2-type immune response. Unexpectedly, the ID route leads to a balanced Th1 and Th2 immune response. In addition, the ID route conferred effective protection against lethal challenge with 40 LD50 of the rabies CVS strain, which was followed by IP and IM routes. Moreover, a one-third dose of the vaccine inoculated via the ID route provided comparable or higher efficacy to a full dose of the vaccine via the other three routes. The superior performance of ID inoculation over other routes is related to longer local retention at injection sites and higher lymphatic drainage. Histopathology examination reveals a transient inflammatory cell infiltration at ID and IM injection sites which peaked at 48 h and 24 h, respectively, after immunization, with all side effects disappearing within one week. These results suggest that needle-free injection technology-based ID inoculation is a promising strategy for rabies vaccination in regard to safety and efficacy.

## 1. Introduction

The inoculation route is of importance in influencing vaccine immune response, which represents a key research area in the field of immunology. There have been various inoculation routes reported in vaccine studies [1,2,3]. Generally, they can be divided into two categories: mucosal and parenteral routes. The former can be further categorized into nasal and oral routes which have been successfully used for licensed cold-adapted influenza vaccines, adenovirus vector-based COVID-19 vaccines and live-attenuated virus vaccines (e.g., poliovirus, typhoid and rotavirus) [4,5]. Vaccines delivered via the mucosal route can induce both mucosal and systemic immune responses. However, this administration route usually requires to use of engineered virus vectors or live-attenuated viruses, which may induce the risk of vector-related pre-existing immune response or vaccine-derived disease due to reversion to virulence, as in the case of polio [6]. Parenteral routes include intramuscular (IM), subcutaneous (SC), intradermal (ID), intraperitoneal (IP) and intrathecal (IT) administrations, which can induce a robust systemic immune response. Among these routes, IM injection is the most frequently used inoculation route for the majority of marketed human vaccines, while SC is the second most commonly used route for vaccination. Only a small part of marketed human vaccines have been recommended for inoculation via the ID route, such as smallpox (scarification/multipuncture), BCG and some rabies and influenza vaccines (needle-assisted injection technique) [7,8,9,10,11]. IP administration is straightforward to handle and can be used to inject a large volume of fluid, but it is only limited to laboratory rodents [12]. IT immunization involves injecting antigens into the subarachnoid spaces and can induce antigen-specific antibodies in serum and cerebrospinal fluid. However, this inoculation route requires a skilled administration technique and has the risk of central nervous system damage [13,14,15]. Until now, there have been no vaccines approved for human use via IP and IT routes, although few clinical trials involved the above routes [16,17].

Among these parenteral routes, ID may be one of the promising routes in view of vaccine efficacy. This is because the ID route ensures a great exposure of delivered antigen in skin which, as the first barrier against pathogenic microorganisms, contains various antigen-presenting cells (APCs) such as Langerhans cells, dermal dendritic cells and dermal macrophages. These APCs are responsible for capturing, processing and presenting antigens to immune effector T cells and meanwhile release chemokines and cytokines to recruit other immune cells to the injection site, therefore facilitating the lymphatic drainage of antigens [18,19]. In a previous clinical study, needle-assisted ID inoculation of hepatitis B, at months 0, 1, 2 and 6, resulted in a higher serum conversion than IM inoculation at the same dose and with the same inoculation schedule [20]. Levin et al. reported that the trivalent inactivated influenza vaccine, containing 3 μg hemagglutinin for each antigen, inoculated with needle-assisted ID injection technology induced higher immunogenicity in humans than that delivered by intramuscular injection of 15 µg hemagglutinin of each antigen [21]. Despite excellent performance in immunogenicity, needle-assisted intradermal vaccine delivery is more complex to execute than IM, SC or IP injections [22]. It requires appropriate staff training and qualified medical supervision because it is challenging for a vaccine to enter into the skin only, without leakage into other tissues, such as subcutaneous tissue. Moreover, for all parenteral routes, needles may induce pain at injection sites, and needle re-use may lead to infectious diseases such as hepatitis B and acquired immunodeficiency syndrome [23]. 

So far, many intradermal delivery devices have been developed to overcome user-dependent issues when delivering cargo into the dermis. These devices include mainly microneedles (MNs) and jet injectors (for high-velocity powder and liquid contents) [24]. MNs are an array of micron-sized needles that can penetrate the epidermis and upper dermal layer of skin. MNs are a minimally invasive method, therefore reducing pain and infection risk and improving user compliance [25]. The MNs marketed as MicronJet600, consisting of three needles of 600 µm length, have been used to administrate inactivated influenza vaccine, inactivated polio vaccine (IPV) and varicella-zoster virus vaccine (VZV) [26,27,28]. In contrast, high-velocity powder and liquid jet injection, as a non-invasive inoculation route, can eliminate needle-related dangers and pain. The majority of jet injectors currently being developed for vaccine delivery are disposable syringe jet injectors (DSJIs) which avoid cross-contamination [24]. The principle of liquid jet injection is to use gas or spring power to force liquid medication to run through a tiny orifice, which creates a liquid high-pressure stream to pierce through skin and deposit medication in the desired location [29]. DSJIs are easy to use and operate, allowing patients to inject on their own anywhere without the supervision of medical staff. In addition, these devices reduce sharp wastes, needle-stick injuries and associated consumable costs. Jet injectors such as the PharmaJet Tropis^®^, Biojector 2000^®^ and MIT Canada Med-Jet Dart^®^ have been evaluated for various seasonal and pandemic flu vaccines, the zoster vaccine, IPV and insulin in a large number of clinical trials [7,28,30,31]. Among these, the PharmaJet Tropis^®^ is the WHO-qualified, needle-free delivery system for an inactivated polio vaccine [32,33].

Rabies is a fatal zoonotic disease of the central nervous system. The rabies virus infects humans via direct contact with virus-carrying animals, such as severe bites. There are approximately 59,000 human deaths due to rabies virus infection every year, and more than 95% of these occur in Asia and Africa [34,35]. Rabies virus infection can be effectively prevented by the implementation of post-exposure prophylaxis (PEP). WHO-recommended PEP includes timely wound washing, rabies vaccination and rabies immunoglobulin injection in case of category III exposures [36,37]. In most of the developed countries, vaccination of domestic animals has significantly reduced rabies cases in humans [38]. In many countries, the commercial rabies vaccine is inoculated via the intramuscular route (IM) with a 0.1 mL dose given three times within one week [39]. For example, India, Philippines, Sri Lanka, Thailand, Pakistan, Nepal, Bangladesh, Madagascar and the United Republic of Tanzania use intradermal inoculation (ID) routes for rabies vaccination [40,41,42,43,44]. In addition, other routes such as IM, IP and SC, have been reported in rabies immunological studies. However, there are a few studies involving systematic head-to-head comparison and investigation of the effect of the abovementioned inoculation routes on vaccine immune response and safety. 

Therefore, the aim of this study is to investigate and compare the immune response and safety of the rabies vaccine inoculated via IP, IM, SC and needle-free injection technology-based ID routes. As far as we know, this work is the first study to perform a side-by-side comparison between these four administration routes. Systemic comparison of the immune response via these inoculation routes was evaluated in terms of antigen-specific antibodies, antibody subtypes and neutralizing antibodies over more than half a year. The protective efficacy of the vaccine was also determined through a lethal rabies virus challenge. In addition, a histopathological assay was performed to illustrate the dynamic migration of inflammatory cell infiltration in the skin and muscle tissues. The change in body weight during 8 weeks after immunization was also examined. In order to reveal the potential mechanism behind the effects of various inoculation routes on immune response, in vivo imaging analysis was conducted to examine the diffusion and dispersion kinetics of antigens at both the local injection site and the lymphatic and metabolic tissues. This study provides valuable information on the effect of immunization routes on the safety and efficacy of the rabies vaccine. This deep insight into the selection of vaccination routes for rabies may be applicable to other vaccines and their clinical inoculation.

## 2. Results

### 2.1. Rabies-Specific Antibody Response

**Figure 1:**
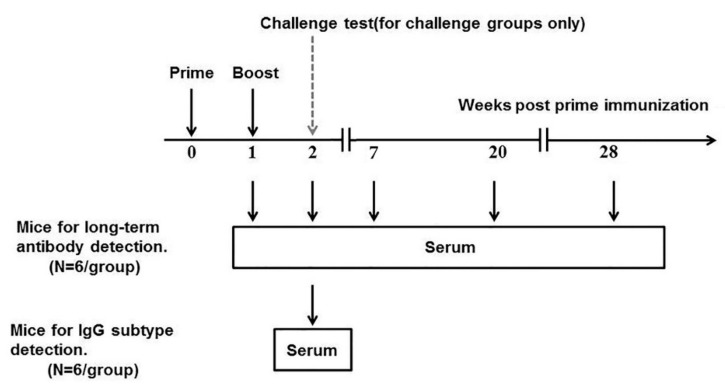
The schematic plan for inoculation of ICR mice with the rabies vaccine. ICR mice (six per group) were inoculated with the rabies vaccine on week 0 and week 1. On weeks 1, 2, 7, 20 and 28, blood samples were collected.

**Figure 2:**
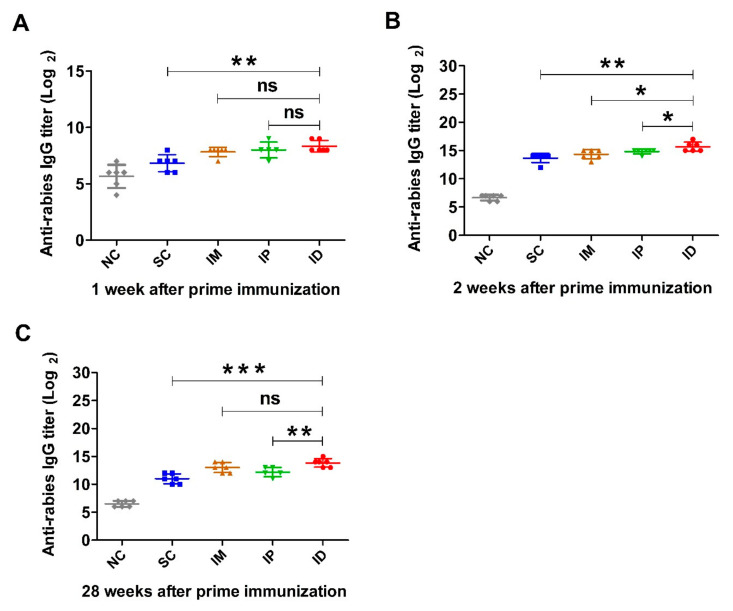
Rabies-specific IgG levels induced by rabies vaccine via various immunization routes. Mice (*n* = 6) were immunized twice at a 1-week interval via SC, IM, IP or ID, and a non-immunization group was used as control. Blood samples were collected at different time points after prime immunization. Rabies-specific IgG titers at weeks 1 (**A**), 2 (**B**) and 28 (**C**) were detected by ELISA. Statistical difference of IgG titer: ns, non-significant; * *p* < 0.05; ** *p* < 0.01; *** *p* < 0.001.

First, rabies-specific IgG levels in serum of mice inoculated with rabies vaccine one week apart (Figure 1) were measured by ELISA, and the results are shown in Figure 2. The antibody titers of control groups at all sampling time points are less than 2^7^. Figure 2A demonstrates that one week after the primary inoculation, all groups immunized with the rabies vaccine produced a rabies-specific IgG antibody response. ID group showed higher titer than IP (*p* = 0.3893) and IM (*p* = 0.0924), although there was no statistically significant difference between them. However, mice immunized via the SC route showed a significantly lower antibody titer than those immunized via IP (*p* = 0.0274), IM (*p* = 0.0169) and ID (*p* < 0.01). One week after boost immunization, IgG levels of each group dramatically increased and reached peaks (Figure 2B), with a similar trend to those at week 1 (ID > IP > IM > SC). In addition, IgG titers for all vaccinated groups remained at high levels until 7 weeks, which was followed by a gradual decrease over time (Supplementary Figure S1). At week 28, mice immunized via ID and IM induced significantly higher antibody levels than those immunized via IP. Mice inoculated via SC still displayed a lower antibody level than those inoculated via ID (*p* = 0.0001), IM (*p* = 0.0031) and IP (*p* = 0.0486) (Figure 2C). 

### 2.2. IgG Subtype Assay

**Figure 3:**
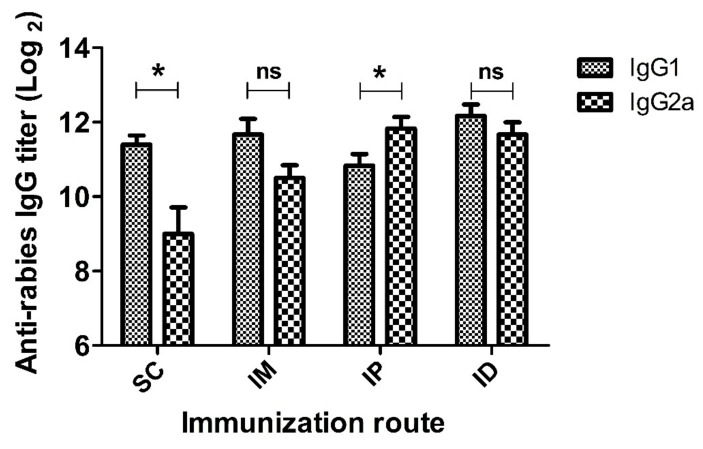
IgG1 and IgG2a titers of mice inoculated with rabies vaccine via SC, IM, IP and ID routes. Mice (*n* = 6) were immunized twice at a 1-week interval. Serum samples were collected at 2 weeks after prime immunization and IgG1 or IgG2a antibody titers against rabies were evaluated by ELISA. Statistical difference of IgG1 titer and IgG2a titer: ns, not significant; * *p* < 0.05.

In addition to rabies-specific IgG titer, IgG antibody subclasses were examined using the ELISA method. The representative result of subclasses at peak IgG level (week 2) is shown in Figure 3. Both SC and IM administrations induced higher IgG1 than IgG2a levels, indicating a Th2-biased immune response. In the case of the IP administration route, IgG2a dominated over IgG1, suggesting a Th1-derived cellular immunity. Interestingly, mice immunized via the ID route exhibited comparable IgG1 and IgG2a levels, indicating a balanced Th1 and Th2 immune response [45]. Overall, these results reveal that administration routes significantly affect both the magnitude and the type of immune response. 

### 2.3. Rabies-Virus-Neutralizing Antibody

**Figure 4:**
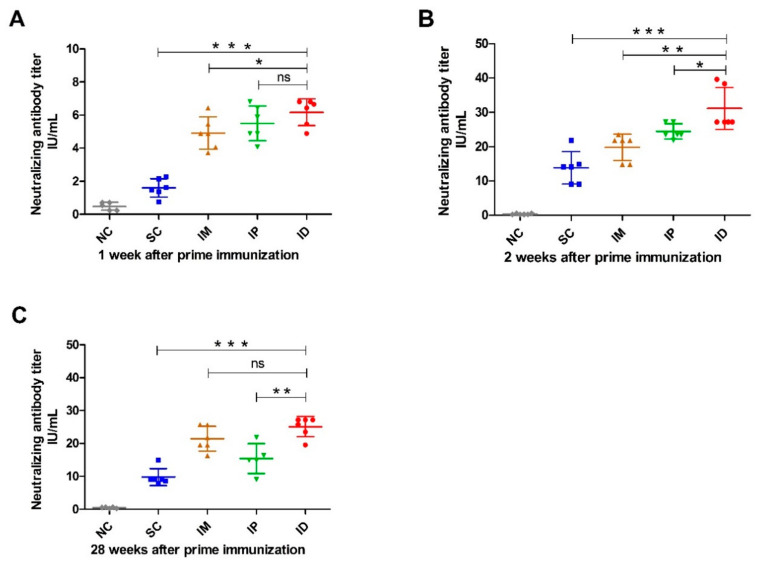
Rabies-virus-neutralizing antibody level in mice inoculated with rabies vaccine via various routes. Mice (*n* = 6) were immunized twice at a 1-week interval via SC, IM, IP and ID routes. Blood samples (*n* = 6) were collected at different time points after prime immunization, and rabies-virus-neutralizing antibody titers on week 1 (**A**), 2 (**B**) and 28 (**C**) were measured using the Rapid Fluorescence Focus Inhibition (RFFIT) test. Titers were expressed in international units/mL (IU/mL) based on the WHO standard. Statistical difference of rabies-virus-neutralizing antibody titers: ns, not significant; * *p* < 0.05; ** *p* < 0.01; *** *p* < 0.001.

In order to further illustrate the effect of the inoculation route on immune response, rabies-neutralizing antibodies were analyzed using RFFIT, and the result is shown in Figure 4. Similar to the result of rabies-specific IgG (Figure 2), all groups inoculated with the rabies vaccine elicited anti-rabies-neutralizing antibodies (RNVAs) one week after primary vaccination. As expected, the ID administration route resulted in higher RNVA levels than other routes (IP and IM groups in Figure 4A). SC administration induced significantly lower RNVA levels than other groups (*p* < 0.05) (Figure 4A). On week 2, the RVNA level in serum significantly increased and reached a plateau for all groups immunized with the rabies vaccine. However, similar trends were shown in RVNA to those after the primary vaccination, giving the order of ID > IP > IM > SC. Of note, the IP group produced a significantly higher level of RVNA than the IM group (*p* = 0.0283) (Figure 4B). The RVNA titers for all vaccinated groups were sustained at high levels and then gradually decreased after week 20 (Supplementary Figure S2). On week 28, RVNA levels were reduced in all immunized groups. The ID and IM groups showed a comparable RVNA level, followed by the IP group, while the SC group showed the lowest RVNA level among all groups (Figure 4C). 

### 2.4. Challenge Test

**Figure 5:**
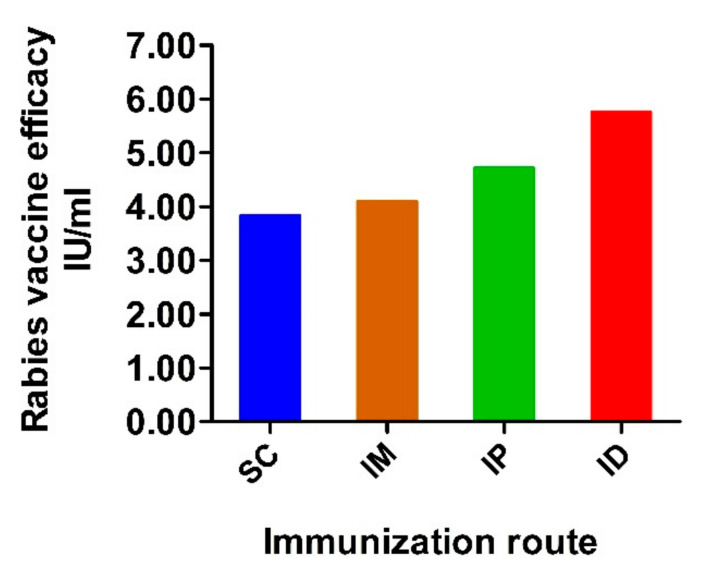
Potency of vaccine administrated via variable routes. Mice were inoculated with rabies vaccine twice at a 1-week interval via SC, IM, IP and ID routes. Two weeks after prime inoculation, mice were challenged with 40 LD50 of CVS11 strain via intracerebral route and observed for another 2 weeks. The efficacy of the rabies vaccine in each group was calculated and expressed as IU/mL.

In addition to rabies-virus-neutralizing antibodies, potency was analyzed according to the National Institutes of Health (NIH) test recommended by the WHO [46]. Mice were immunized on weeks 0 and 1 with 0.5 mL/dose and challenged with pathogenic rabies virus strain CVS11 via an intracerebral route on week 1 after boost immunization. Mice were monitored for the development of rabies for another 2 weeks. The vaccine efficacy of each group is shown in Figure 5. According to the WHO recommendation, a rabies vaccine for human use should have a minimum potency of 2.5 IU per dose after exposure to the rabies virus [47,48]. In our challenge test, all experimental groups met the given standards. Moreover, ID administration induced the highest potency (5.8 IU), followed by IP (4.7 IU) and IM (4.1 IU). Lower potency was observed in the group immunized via the SC route (3.8 IU).

Considering variable immune response and potency resulting from different administration routes, we further evaluated the effect of doses (1 dose, 1/3 dose and 1/9 dose) on the potency of the rabies vaccine. For each administration route, mice inoculated with the rabies vaccine showed dose-dependent protection (Supplementary Figure S3). Under the same dose of immunization, ID administration had a higher level of protection than IP and IM. As expected, SC administration showed the lowest potency. The 1/3 dose vaccine administered via ID induced higher efficacy than vaccination via SC and IM with 1 dose. These results indicate that the inoculation route significantly influences vaccine potency and that the ID route can provide comparable or higher protection against CVS11 attacks than other administration routes even at lower doses.

### 2.5. Histopathological Analysis and Body Weight

**Figure 6:**
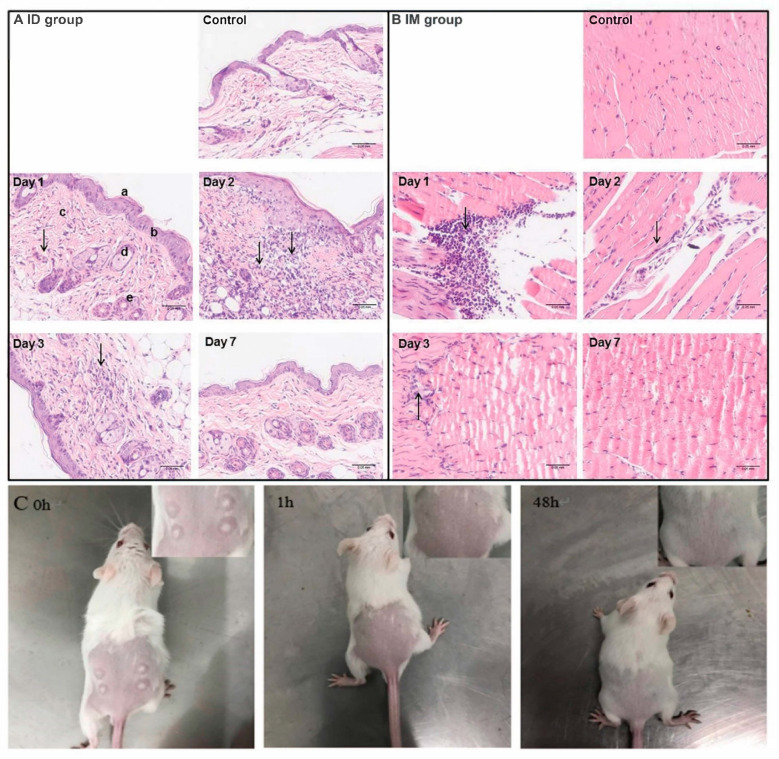
Histological changes in skin tissue (**A**) and muscle tissue (**B**) at the injection sites in mice at different time points after inoculation. (**C**) The typical appearances after needle-free injection technology-based intradermal (ID) inoculation. The arrows shown in (**A**,**B**) indicate the infiltration of inflammatory cells. Scale bar is 0.05 mm.

In order to investigate the local inflammatory response that occurred in skin and muscle following ID and IM administration routes, hematoxylin and eosin staining was conducted on days 1, 2, 3 and 7 after inoculation (Figure 6). Measurements of local inflammatory responses for SC and IP routes were also attempted. However, no reliable results were available due to the fast diffusion of the vaccine in injection sites. For ID administration (Figure 6A), mild infiltration of inflammatory cells was found throughout the dermis and subcutaneous fat on day 1, as shown by the presence of an increasing amount of blue (nuclear) staining compared to that of the control group. The number of infiltrating inflammatory cells was significantly increased on day 2, followed by a decrease on day 3. Furthermore, the ID group presented slight hyperkeratosis and epidermal hyperplasia over 3 days. On day 7, there were no significant differences in inflammatory cells between ID and control groups, suggesting a complete recovery from ID route inoculation. For IM administration (Figure 6B), severe inflammation infiltration was observed on day 1 upon injection of the rabies vaccine. However, the local inflammatory response was dramatically decreased on day 2. Damaged muscle fibers were found to have been completely restored, and additional inflammatory response disappeared one week after inoculation. An interesting phenomenon is that intramuscular inoculation had an earlier transient inflammatory response at injection sites than intradermal injection. This may be because intramuscular injection causes more severe tissue damage and faster blood circulation in muscle recruits more inflammatory cells. Additionally, for ID administration (Figure 6C), the appearance change of skin at injection sites was recorded. The skin of mice in this group developed a white blister with a micropore in the center caused by the gas-driven liquid high-pressure stream. However, the white blister disappeared within 1h after immunization and did not affect the hair growth of mice. Lastly, the body weight of each group was recorded over 8 weeks following immunization via various routes (Supplementary Figure S4). All mice gained weight gradually over time. There was no significant difference between vaccinated and control groups, suggesting no remarkable influence of administration routes on mouse growth.

### 2.6. In Vivo and Ex Vivo Imaging Analysis

**Figure 7:**
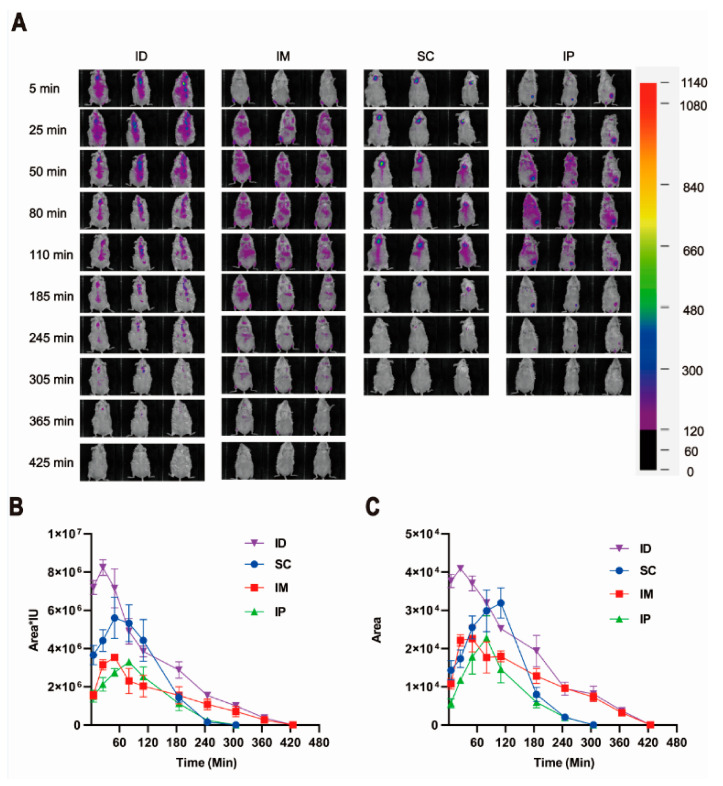
Local retention and diffusion kinetics of model antigen Cy7-OVA in ICR mice injected via various routes. (**A**) Representative in vivo images of three mice injected with the same amount of Cy7-OVA (fluorescence intensity of 1440 in Eppendorf Tube^®^) by intradermal (ID), intramuscular (IM), subcutaneous (SC) and intraperitoneal (IP) routes. (**B**) Relative fluorescence radiance of Cy7-OVA via various inoculation routes shown as fluorescent region area multiplied by average fluorescence intensity. (**C**) Retention and diffusion kinetics of model antigen Cy7-OVA at injection sites shown as the fluorescent regional area around injection sites.

We first investigated the local diffusion kinetics of antigens at the injection site by replacing the rabies vaccine with Cy7-conjugated OVA. As shown in Figure 7A,C, all groups showed a gradual diffusion of Cy7-OVA over time after inoculation, which was followed by elimination at different rates. Among all injection routes, the ID group showed the largest distribution. Interestingly, the retention of the model antigen at the local injection site in the ID group was still observable at 365 min after vaccination, which was comparable to that in the IM group (Figure 7A,C), suggesting a slow elimination. In contrast, both the SC group and IP group lasted only up to 245 min. Both IM and IP groups showed an overall lower fluorescence radiance at both local and systemic dispersion sites than ID and SC groups in the first 180 min after injection (Figure 7B). This may be explained by injection depth and the resultant tissue penetration and absorption which can cause a negative effect on the NIR signal (such as both excitation light and emission light) [49]. Indeed, in both IM and IP groups, the model antigen was injected significantly deeper than that in the ID and SC groups.

**Figure 8:**
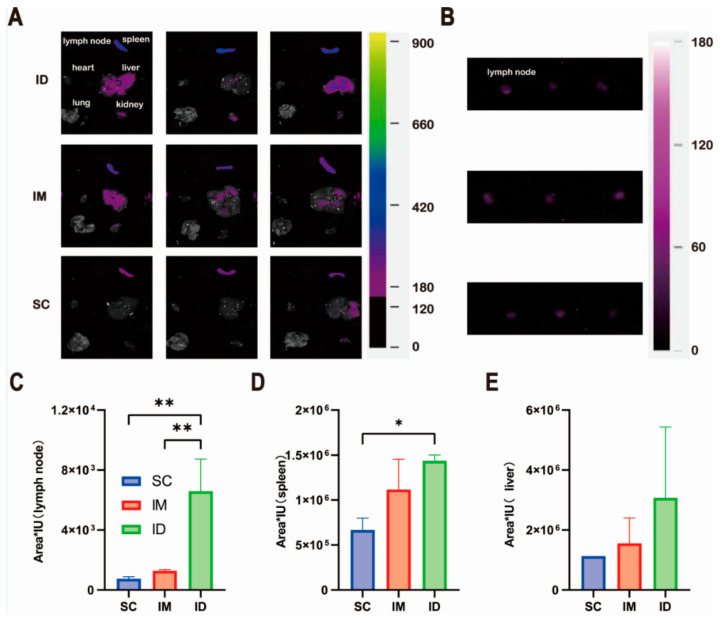
Tissue distribution of model antigen Cy7-OVA in ICR mice injected with the same amount of Cy7-OVA (fluorescence intensity of 1140 in Eppendorf Tube^®^) via intradermal (ID), intramuscular (IM) and subcutaneous (SC) routes: (**A**) images of representative tissues, (**B**,**C**) lymph node, (**D**) spleen and (**E**) liver. All tissues were collected at peak fluorescence radiance in in vivo imaging experiments. All fluorescence images were collected using a FOBI^®^ Imaging System (NeoScience Co., Ltd., Seoul, Republic of Korea) via a near-infrared (NIR) channel with an excitation wavelength of 730 nm. The exposure time was set to 1.05 s (**A**) and 8.00 s (**B**), with the gain value of 10 for all groups. Statistical difference of fluorescence radiance: * *p* < 0.05; ** *p* < 0.01.

In order to illustrate the distribution of the model antigen, we further investigated the peak fluorescence radiance of the model antigen in major ex vivo tissues. Considering the relatively weak fluorescence signal, the images of draining lymph nodes near the injection sites in each group were further collected using a longer exposure time of 8 s instead of 1.05 s as shown in Figure 8. It is worth noting that the IP group was not displayed and compared with other groups as it is difficult to distinguish the distribution of model antigens in various tissues from their adsorption on the surface of each tissue. Figure 8A,B show that model antigen was detectable in the liver, spleen, kidney and draining lymph nodes near the injection sites, but not heart or lung, for all groups. The ID group shows the highest antigen distribution in both the lymph nodes and the spleen (Figure 8C,D), while no statistically significant difference was found in the liver between the groups (Figure 8E).

## 3. Discussion

Although other non-neutralizing antibodies, cell-mediated immunity or interferons may also contribute to the effective clearance of the virus from both the bite site and the central nervous system [50,51], protection against rabies virus infection resulting from vaccination is closely related to the primary role of neutralizing antibodies to prevent the virus from attaching to neurons [52]. Early antibody response and long-term antibody persistence are essential for post-exposure immunization. The generation of an early and robust antibody response, especially a neutralizing antibody response, is beneficial for clearing the virus during the early infective stage, while a high and long-lasting antibody level, especially the neutralizing antibody level, may provide longer protection against severe infection or reinfection. In this study, we found that the intradermal route elicited comparable rabies-specific antibody and neutralizing antibody levels to the intraperitoneal route after primary vaccination, and it was followed by the intramuscular route (Figure 2 and Figure 4). The subcutaneous route showed the lowest antibody levels, which agrees with the study reported by Wunderli et al. [3]. In our previous studies investigating the immunogenicity of zein nanoparticles, the subcutaneous inoculation group also showed a suboptimal humoral immune response when compared with the intramuscular inoculation group [53]. The difference in immune response coming from inoculation routes is most likely related to varied APC distributions in each injection site, which lead to differences in antigen-presenting efficiency. The abdominal cavity is filled with the intestine and other organs. The intestine is known as the largest immune organ in the body for mammals including mice and contains various immune tissue and cells [54,55], while skin is known to have a widespread network of APCs (e.g., Langerhans cells, dermal dendritic cells and dermal macrophages) and extensive connections to regional lymph nodes [36,56,57]. In the case of the subcutaneous tissue of mouse skin, less APC distribution has been reported [36,58], which partly explains why the immune response elicited via subcutaneous injection is least effective. 

Besides APC distribution, the local diffusion, lymphatic drainage and distribution of antigens in lymphatic tissue may also influence the subsequent immune response. In order to verify the above hypothesis, in vivo imaging analysis was performed to illustrate the local diffusion kinetics of the model antigen at injection sites. Among all groups, the largest distribution in the ID group is probably due to multipoint injection performed to mimic the real-life situation of the above vaccination in which only a limited volume per site can be accommodated in mouse skin (Figure 7). It has been reported that multipoint injection may provide more chances for antigens to contact APC cells and therefore may lead to higher antigen presentation efficiency [59]. The faster antigen diffusion at the local injection site in the SC group is most likely due to a less dense physiological structure and more abundant capillary distribution in subcutaneous tissue. In the case of the IP group, large space in the abdominal cavity, the presence of abundant intestinal capillaries, and metabolism and excretion organs such as the liver and kidney directly lead to the rapid spread and elimination of antigens. Rapid and widespread antigen diffusion at the local injection site means the escape of antigens from the injection-injury-induced local immune microenvironment and therefore may be detrimental to antigen uptake and presentation by recruited immune cells due to spatiotemporal misalignment. The reasonable speculation is supported by the fact that ID inoculation prolonged the deposition of the antigen at the site, and therefore enhanced its uptake by antigen-presenting cells [1]. It is worth noting that the presence of a large amount of lymphoid tissue along the intestine partially counteracts the above negative effects for the IP group, but the relative lack of subcutaneous lymphoid tissue may further exacerbate these negative effects for the SC group. Despite this, both ID and IM groups still displayed better antibody persistence than the IP group as evidenced by the high rabies-specific antibody and neutralizing antibody levels at week 28 after primary inoculation (Figure 2 and Figure 4). The inferior performance of the intraperitoneal route in long-term antibody persistence in comparison with intramuscular injection has also been observed in BALB/c mice inoculated with inactivated influenza vaccine in another ongoing study of our group. 

The specific distribution of the model antigen in the liver, spleen, kidney and draining lymph nodes, but not the heart or lung (Figure 8), can be explained by the physiological structural features of each organ. The liver, spleen and lymph nodes are rich in sinusoidal capillaries with high permeability to large molecules, while the kidney is full of fenestrated capillaries with pore sizes smaller than those of sinusoidal capillaries [60]. Therefore, these organs allow the penetration of the migrated model antigen from the injection site and give a fluorescence signal. Among all organs, the distribution of the model antigen in the draining lymph nodes and spleen is essential to induce a strong and durable immune response [61,62]. Thus, more distribution in the draining lymph nodes and spleen is favorable for a stronger immune response (Figure 8), which is supported by antigen-specific antibody levels and antibody persistence (Figure 2, Figure 4 and Figure 5). In summary, a few factors such as high APC density, prolonged deposition at injection sites and high lymphatic tissue dispersion altogether contribute to better performance of intradermal inoculation in antibody production and persistence than other routes.

Another interesting finding is that administration routes have an impact on immune response type. Although neutralizing antibody is usually used as the principal immunological correlate of protection, balanced humoral and cellular immune responses are more likely to provide comprehensive protection, especially when the virulent viruses start to replicate at wound sites or to spread to neurons. A neutralizing antibody is effective in neutralizing an extracellular virus, but is less likely to clear an intracellular virus [63]. Lebrun et al. have reported that a strong Th2 response in mice did not guarantee survival when the rabies virus reached central nervous tissues [64]. The delay in stopping the virus from spreading from the periphery to the central nervous system will irretrievably lead to the development of rabies. From this perspective, intradermal inoculation is more advantageous than other administration routes as it induces a balanced Th1 and Th2 immune response (Figure 3). A similar phenomenon has also been observed in a study where an inactivated influenza virus vaccine delivered by intradermal dissolving microneedle induced comparable humoral and cellular immunity in BALB/c mice [65]. Although the bias of immune response can be influenced by various factors, such as antigen epitope [66], adjuvant formulation [67] and animal species [68], the abovementioned difference in immune response type in our study is mainly due to the immunization route as other factors are not involved. Intradermal inoculation can directly activate the innate dendritic cell network and further lead to the activation of an adaptive immune response, which may account for the superb immune magnitudes and types [19]. It should be noted that the inferior immune response and Th2-biased immune response type of the subcutaneous route in mice may not reflect the situation in humans as the skin of fur animals like mice is more loosely attached to muscle tissue and has a different anatomical structure from human skin [69]. 

## 4. Materials and Methods

### 4.1. Vaccine

The human rabies virus vaccine (inactivated) was provided by Changchun BCHT Biotechnology Co., Ltd. (Changchun, China). The potency of the vaccine was above 2.5 IU/mL.

### 4.2. Animals

Female ICR mice (18–20 g) were purchased from the Changchun Institute of Biological Products and maintained under specific-pathogen-free conditions. The mice were housed under a 12 h light/dark cycle. Feed and water were supplied ad libitum. All animals were treated according to the Guide for the Care and Use of Laboratory Animals (National Research Council, Washington, DC, USA). All experimental procedures were reviewed and approved by the Animal Welfare and Research Ethics Committee of the School of Life Sciences at Jilin University (Ethical approval number 2022—YNPZSY047).

### 4.3. Immunization and Bleeding

To determine the effect of inoculation routes on rabies immunogenicity, thirty female ICR mice were randomly divided into five groups (six mice per group). Four groups of mice were injected twice with 0.5 mL/dose of rabies vaccine via intraperitoneal, intramuscular, subcutaneous and needle-free injection technology (POK-V-MBX Needle-free Injector, Jiangsu Poke Medical Technology Co., Ltd., Taizhou, China)-based intradermal routes, respectively. For the intramuscular route, the rabies vaccine was inoculated at 4 sites of the front and hind thighs (0.125 mL per site). For the intradermal route, the rabies vaccine was inoculated at 10 dorsal sites (0.05 mL per site). Unimmunized mice were used as controls. Booster vaccines were given one week after the first dose of primary vaccination. The body weight of mice in each group was detected until the eighth week after the first immunization. Blood samples were collected from the orbital venous sinus at scheduled time points, and the serum was separated by centrifugation twice (3000 rpm, 20 min) and kept at −80 °C until use.

### 4.4. Measurement of Rabies-Specific IgG and Subclass

An indirect ELISA was conducted to detect serum rabies-specific IgG according to the manufacturer’s instructions. Briefly, 96-well ELISA plates were coated with 100 µL inactivated rabies vaccine without human serum albumin diluted in pH 9.6 carbonate-bicarbonate buffer overnight at 4 °C. The plates were then washed three times with phosphate-buffered saline containing 0.05% Tween-20 (PBS-T) and blocked with 200 µL of 1% BSA-PBS (*w*/*v*) at 37 °C for 2 h. After another washing step, the sera (100 µL) were diluted in 2-fold series with blocking buffer in eight consecutive wells of the plate and incubated at 37 °C for 1 h. The plates were washed three times with PBS-T and 100 µL diluted peroxidase-conjugated anti-mouse IgG (Dingguo Changsheng Biotechnology. Co., Beijing, China); anti-mouse IgG1 or anti-mouse IgG2a (Sigma-Aldrich, St. Louis, MO, USA) was then added to plates and incubated at 37 °C for 1 h. For the isotype antibody assay, the plates were washed as above, and 100 µL of diluted peroxidase-labeled rabbit anti-goat IgG (Dingguo Changsheng Biotechnology. Co., Beijing, China) in blocking buffer was added to each well for 1 h. After washing three times, 50 µL 3,3′,5,5′-tetramethylbenzidine substrate was added to each well, and the plate was incubated in the dark for 30 min at room temperature. The reaction was stopped by adding 50 µL/well of H_2_SO_4_ (2 M). The optical density of the plate was read at 450 nm using an automatic microplate reader (Bio-Rad Laboratories, Inc., Hercules, CA, USA). The antibody titer was expressed as the log 2 value of serum maximum dilution whose OD value was 2-fold higher than the average OD of the blank group (negative control, NC).

### 4.5. Measurement of Neutralizing Antibodies

The measurement of rabies-virus-neutralizing antibodies (RVNAs) in serum was performed using a rapid fluorescent focus inhibition test (RFFIT) as described previously [70]. The national reference standard serum and pre-immune serum were used as positive and negative controls, respectively. Briefly, 50 µL samples of serial 3-fold dilutions of test and standard serum using Dulbecco’s Modified Eagle Medium (DMEM) with 2% fetal bovine serum (FBS) were mixed with 50 µL of the rabies virus CVS11 strain (4 × 10^7^ FFU/1.0 mL, provided by ATCC) in 96-well tissue culture plates. The plates were incubated at 35–37 °C in a humidity incubator with 5% CO_2_ for 1 h. Thereafter, 50 µL of BSR cell suspension, containing 5 × 10^5^ cells per milliliter, was added into each well, and the plate was returned to the inoculator for another 24 h. After incubation, the culture medium in each well was thrown away, following which the cells were fixed with 80% ice-cold acetone (50 µL per well) for 30 min at 4 °C. Subsequently, the cells were stained with FITC-conjugated anti-rabies nucleoprotein antibody (Light Diagnostics ^TM^, CA, USA) for 1 h at 37 °C in the dark and then washed three times with PBS. Numbers of fluorescent foci that represented the percentage of cell infection were counted using an Olympus IX51 fluorescence microscope. The international unit (IU) of rabies-virus-neutralizing antibodies of tested sera was calculated based on a comparison to the national reference standard serum using the Reed and Muench method [71].

### 4.6. Protective Efficacy against Rabies Virus CVS Challenge

The challenge studies were conducted in accordance with the National Institutes of Health (NIH) efficacy test for rabies vaccine. International reference standard rabies vaccine with the potency of 6.6 IU/mL, rabies virus standard strain (CVS) and reference standard serum were supplied by the National Institute of the Pharmaceutical and Biological Products Control, China. The median lethal dose (LD50) and median tissue culture infectious dose (TCID50/mL) of the CVS strain were determined. Human rabies virus vaccine (inactivated) provided by Changchun BCHT Biotechnology Co., Ltd. (Changchun, China), was used as the experimental vaccine. Both the experimental vaccine and international reference standard rabies vaccine were diluted in a serial 5-fold dilution (1/25, 1/125, 1/625). For each administration route, 0.5 mL of the undiluted experimental vaccine and each diluted experimental vaccine were injected into sixteen female mice via the corresponding inoculation route on day 0 and 7, while the undiluted and each diluted international reference standard rabies vaccine were injected into sixteen female mice via the intraperitoneal route. All mice were then challenged on day 14 via an intracerebral administration (I.C.) of 30 µL rabies strain CVS11 containing 40 LD50. Subsequently, mice were observed for another 14 days, and the mortality of mice was recorded to calculate the ED50 that is normalized with international reference standard vaccine using the Reed and Muench method to obtain a titer in IU NIH/dose [72]. The relative potency of the experimental vaccine to the international reference standard vaccine for each administration route was determined by comparing the ED50 of the experimental vaccine with that of the international reference standard vaccine.

### 4.7. Histopathological Analysis

To determine inflammatory response at injection sites, mice were randomly divided into two groups with eight in each group and were inoculated by ID and IM injections, respectively. A volume of 100 µL was injected into the left thigh (IM) and the dorsal skin (ID) of each mouse at day 0. Another two naïve female ICR mice were used as a control group. Two mice in each group were sacrificed on days 1, 2, 3 and 7 after immunization. Inoculation sites (about 1 cm^2^ in size) were dissected at indicated days, fixed with 4% paraformaldehyde and embedded in paraffin. The sections were stained following a published hematoxylin and eosin procedure [73]. The slides were visualized using an Olympus CX31 microscope, and the number of inflammatory cells was examined using Photoshop CS4 analysis software.

### 4.8. In Vivo and Ex Vivo Imaging Analysis

To illustrate different diffusion kinetics of the inactivated rabies vaccine after inoculation via various routes, Cy7-OVA (Ruixi Biological Technology Co., Ltd., Xi’an, China) was used as a model antigen, and female ICR mice (at least three mice per group) were injected with the same amount of Cy7-OVA in the same volume (50 µL) via IP, IM, SC and ID routes. To avoid interference, mouse hair from and around injection sites was removed by using clippers and then applying a layer of depilatory creams followed by washing with water. Mice were anesthetized by inhalation of isoflurane (2–4%, *v*/*v*) for 4–5 min at indicated time points, and in vivo fluorescence images were collected using a FOBI^®^ Imaging System (NeoScience Co., Ltd., Seoul, Republic of Korea) via a near-infrared (NIR) channel with an excitation wavelength of 730 nm. The exposure time was set to 1.05 s, and the gain value was set to 10. In order to illustrate the accurate distribution of the model antigen in various organs, mice were sacrificed at indicated time points, and the lymph node, heart, liver, spleen, lung and kidney were collected and imaged using a FOBI^®^ Imaging System (NeoScience Co., Ltd., Seoul, Republic of Korea). The resultant in vivo and ex vivo images were analyzed using NEOimage software (NeoScience Co., Ltd., Seoul, Republic of Korea). The data of fluorescence radiance were shown as the fluorescent region area multiplied by the average fluorescence intensity. The area means the number of pixels within the designated region of interest.

### 4.9. Statistical Analysis

Statistical analysis was conducted using a two-tailed unpaired Student’s *t*-test using GraphPad Prism (version 5.0, GraphPad Software, CA, USA). Data are presented as mean ± standard deviation (SD). The comparison between groups was considered statistically significant if *p* < 0.05.

## 5. Conclusions

In this study, the immune response and safety of the rabies vaccine were systematically evaluated in mice via intraperitoneal, intramuscular, subcutaneous and needle-free injection technology-based intradermal routes, respectively. The intradermal route elicited the highest IgG and neutralizing antibody titers among all injection routes. Interestingly, different routes were observed to show different immune response types. For example, in our studies, the intradermal route induced a balanced Th1/Th2 immune response, while intramuscular and subcutaneous routes induced a Th2-biased immune response, and the intraperitoneal route induced a prominent Th1-type immune response. In addition, a 1/3 dose of the vaccine inoculated via needle-free intradermal injection technology can achieve an efficacy comparable to or higher than that of a full dose via other routes. This significant effect on the immune magnitude and type highlights the pivotal role of antigen delivery via different administration routes, which may be explained by varied APC cell distribution, antigen diffusion, lymphatic drainage and distribution of antigens in lymphatic tissue. This study suggests that the needle-free injection technology-based intradermal route may be an optimal choice for rabies vaccine. Further studies at cellular and molecular levels are required to illustrate the underlying mechanism of varied immune responses induced by different administration routes.

## Data Availability

The data presented in this study are available upon request from the corresponding author.

## Supplementary Materials

The supporting information can be downloaded at: https://www.mdpi.com/article/10.3390/ijms241713587/s1.
