# Supplementary material for: Immune Response of Inactivated Rabies Vaccine Inoculated via Intraperitoneal, Intramuscular, Subcutaneous and Needle-Free Injection Technology-Based Intradermal Routes in Mice
Source: Int J Mol Sci. 2023 Sep 2;24(17):13587. doi: 10.3390/ijms241713587 (PMC10488038; doi:10.3390/ijms241713587)
Supplement: Supplementary file 1 [file ijms-24-13587-s001.zip › ijms-2579898-supplementary.pdf]

Immune Response of Inactivated Rabies Vaccine Inoculated via Intraperitoneal, Intramuscular, Subcutaneous and Needle-Free Injection Technology-Based Intradermal Routes in Mice

Huiting Zhao [1,2,†], Peixuan Li [1,2,†], Lijun Bian [1,2], Wen Zhang [1,2], Chunlai Jiang [1,2,3], Yan Chen [1,2,3,*], Wei Kong [1,2,3] and Yong Zhang [1,2,3,*]

[1] National Engineering Laboratory for AIDS Vaccine, School of Life Sciences, Jilin University, Changchun 130012, China; 3120205689@bit.edu.cn (H.Z.); pxli21@mails.jlu.edu.cn (P.L.); bianlj21@mails.jlu.edu.cn (L.B.); zhangwen21@mails.jlu.edu.cn (W.Z.); jiangcl@jlu.edu.cn (C.J.); weikong@jlu.edu.cn (W.K.)

[2] Key Laboratory for Molecular Enzymology and Engineering of Ministry of Education, School of Life Sciences, Jilin University, Changchun 130012, China

[3] NMPA Key Laboratory of Humanized Animal Models for Evaluation of Vaccines and Cell Therapy Products, Jilin University, Changchun 130012, China

* Correspondence: zhypharm@jlu.edu.cn or zhangyongking1@gmail.com (Y.Z.); chen_yan@jlu.edu.cn (Y.C.); Tel.: +86-431-85167751 (Y.Z. and Y.C); Fax: +86-431-85167674 (Y.Z. and Y.C.)

† These authors contributed equally to this work.

Supplementary

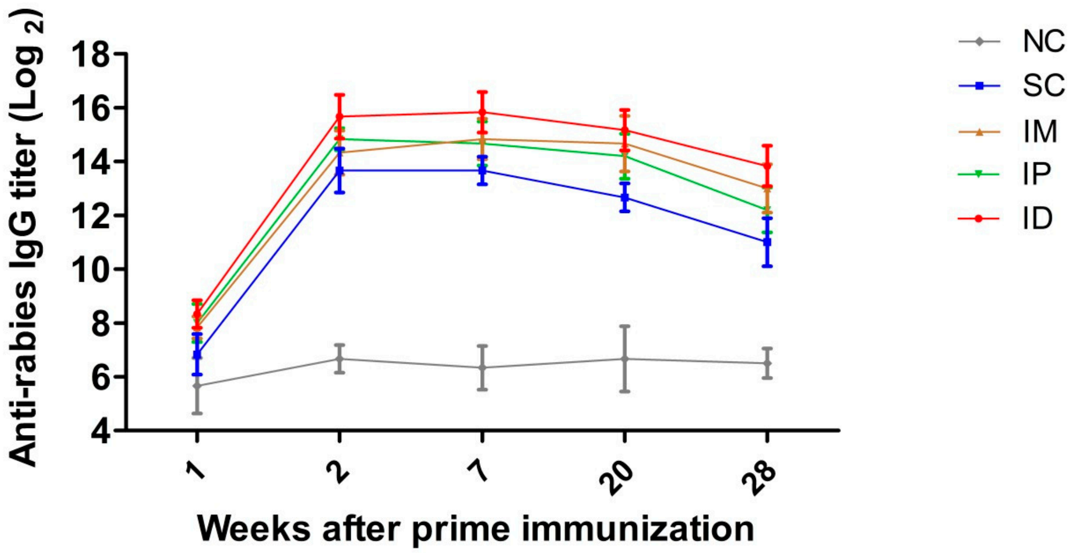

Figure S1. Rabies-specific IgG level induced via various immunization routes. Mice (n = 6) was immunized twice at 1-week intervals via SC, IM, IP or ID, non- immunization as control. Blood samples were collected at at different time after the prime immunization and the kinetic of rabies- specific IgG titers was measured by ELISA.

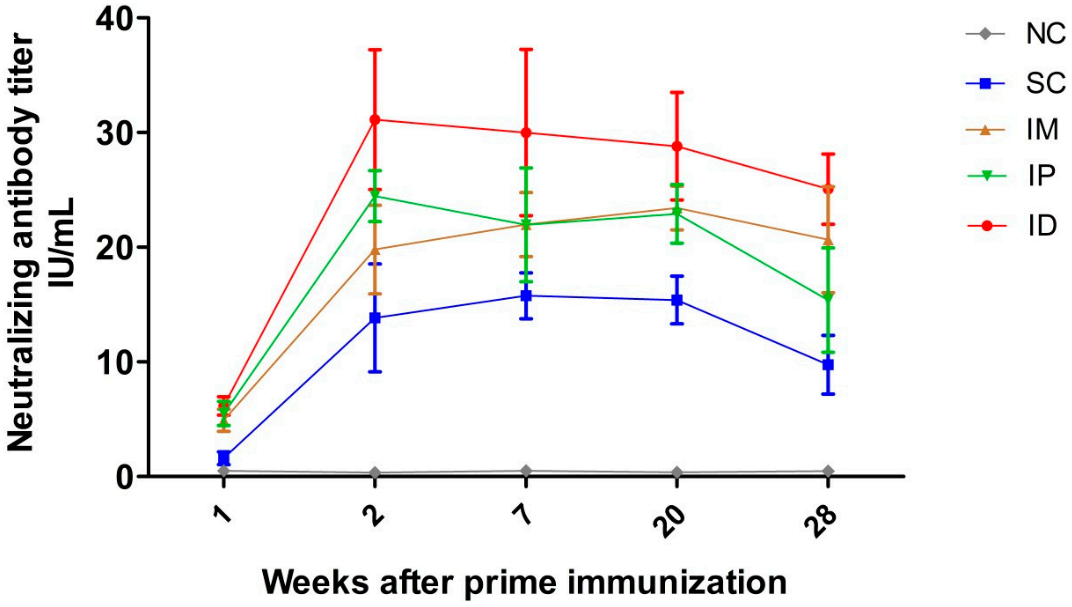

Figure S2. Production of rabies virus neutralizing antibodies in mice following the inoculation of rabies vaccine via SC, IM, IP, and ID route. Mice (n = 6) were immunized twice at 1-week intervals via virious routes. Blood samples (n = 6) were collected at at different time after the prime immunization and the kinetic of rabies virus neutralizing antibodies titers was determined using Rapid Fluorescence Focus Inhibition (RFFIT) test. Titers were expressed in international units/ml (IU/ml) based on the WHO standard.

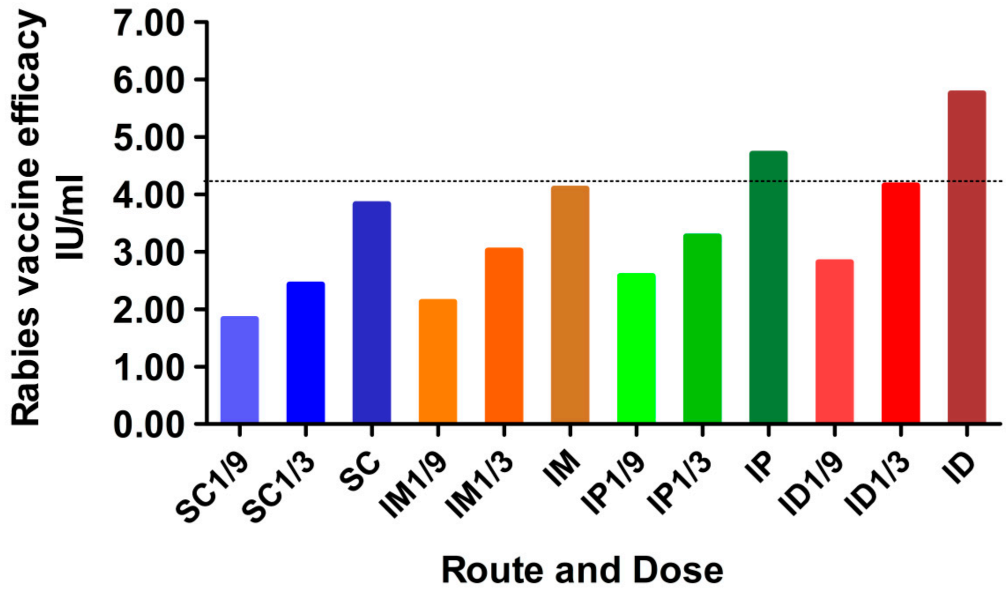

Figure S3. Potency of vaccine administrated via varied routes. Mice were inoculated with 1 dose, 1/3 dose and 1/9 dose rabies vaccine twice at 1-week intervals via SC, IM, IP and ID route. Two weeks after the prime inoculation, the mice were challenged with 40 LD50 of CVS11 strain via intracerebral route and observed survival for another 2 weeks. The efficacy of rabies vaccine in each group was calculated as compared with international reference standard vaccine inoculated via IP route and expressed as IU/ml.

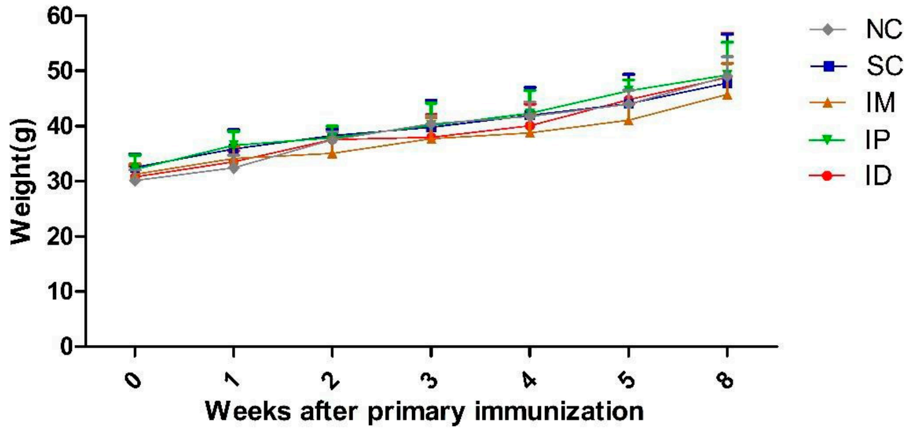

Figure S4. Change of body weight in mice. Mice (n = 9) were immunized twice at 1-week interval via SC, IM, IP or ID routes, and non-immunization group was used as negative control (NC). Body weight change was monitored and recorded for 8 weeks post prime immunization. The data was presented as mean body weight ± SD. No statistical difference was found in body weight between vaccinated and control groups.
